# The high colon cancer risk in African Americans can be reduced by dietary modification

**DOI:** 10.1186/2049-3002-2-S1-O22

**Published:** 2014-05-28

**Authors:** Stephen O’Keefe

**Affiliations:** 1 University of Pittsburgh, PA 15134, USA

## 

Geographical and migration epidemiological studies, backed up by experimental studies, have produced overwhelming evidence that diet drives colon cancer risk, with high intakes of meat and fat increasing risk, and high consumption rates of fiber rich foods, such as grains, fruits and vegetables, reducing risk. To test our hypothesis that cancer risk is determined by the effect the diet has on the composition and function of the microbiota to produce metabolites that either promote mucosal health or are inflammatory and neoplastic, we have conducted studies in populations of high and low risk, namely African Americans who have the highest risk in the USA, and rural Africans, who rarely get the disease, and confirmed that high meat and fat intakes in Americans were associated with increased mucosal proliferation rates – a bio-marker of cancer risk, while high fiber African diets were associated with low proliferation rates. We have demonstrated that dietary switch between these two populations produces reciprocal changes in the microbiome, metabolome, and mucosal biomarkers of risk within 2 weeks, leading to the conclusion that modification of the diets of westernized populations to contain high fiber (>45g/d) foods, such as fruits, vegetables, legumes and grains, can be expected to immediately reduce cancer risk in African Americans and in all high risk westernized populations.

